# Metabolite profiling and transcriptome analyses provide insight into the regulatory network of graft incompatibility in litchi

**DOI:** 10.3389/fgene.2022.1059333

**Published:** 2023-01-05

**Authors:** Yanjie Hou, Xianquan Qin, Hongye Qiu, Dongbo Li, Ning Xu, Shuwei Zhang, Chen Fang, Hongli Li

**Affiliations:** Horticultural Research Institute, Guangxi Academy of Agricultural Sciences, Nanning, Guangxi, China

**Keywords:** litchi, graft incompatibility, productivity, gene expression, metabolome

## Abstract

Litchi is an important commercial fruit crop widely grown in the world. Graft incompatibility between rootstocks and scions is a major constraint for large-scale cultivation of litchi orchards, popularization of new and excellent litchi varieties, and associated industrial development. Further, the genetic mechanism of graft incompatibility is still unclear in litchi. To reduce the incompatibility problems, this study investigated metabolic and transcriptomic differences between graft compatible and incompatible rootstock-scion combinations of litchi. The result of metabolomics analysis showed that incompatible rootstock-scion interaction modified the profiles of several metabolic substances. However, various compounds of flavonoids, phenolic acids, and lignin predominantly exhibited significantly altered abundance in graft incompatible combinations. Transcriptome analysis identified that graft incompatibility induces dynamic gene differences. The majority of these differentially expressed genes were enriched in biosynthetic pathways of phenylpropanoids. The differential expressions of genes in these pathways could be linked to the differential abundance levels of flavonoids, phenolic acids, and lignin compounds. Integrated metabolomic and transcriptomic analyses revealed a strong relationship between differential genes and differential metabolites identified in this study. In addition, identified hub genes and metabolites were closely associated with graft incompatibility of litchi. This study characterized the abundance of metabolites and genes in graft incompatible combinations and further discussed the genetic mechanism of graft incompatibility in litchi. Our results provide a platform to dissect the molecular mechanisms of graft incompatibility in the litchi fruit.

## 1 Introduction

*Litchi chinensis,* belongs to the Sapindaceae family, commonly known as litchi, a native fruit in Southern China. Litchi is widely cultivated in tropical and sub-tropical parts of China for more than a hundred years (Chen et al., 2017). Despite its highly perishable nature, litchi has a great commercial value with an exceptional flavor. It is widely cultivated in more than 20 countries worldwide. China grabs first place in the international market by supplying 60% of the world’s litchi production followed by India, Thailand, and Vietnam (Pareek, 2016). Since genetic variability discourages seedling and cutting propagation in most fruit trees, clonal propagation is practiced in litchi since ancient times. Air layering was well established practice utilized for the propagation of litchi (Das and Birendra, 2014). However, air layering is now replaced by top grafting to improve the inefficiency of rootstock selection programs (Goldschmidt, 2014; Chen et al., 2016). Grafting is the most powerful asexual propagation technique widely performed in perennial fruit such as citrus, figs, apricot, apples, pears, litchi, quince, plum, peach, and grape to improve several agronomic traits such as yield, quality as well as tolerance to biotic and abiotic stresses (Mohamed et al., 2014; Rasool et al., 2020). In addition, its successful application eliminates the juvenile phase and modifies plant growth, devolvement size as well as aroma, and nutrient composition in fruits (Ruiz et al., 2010; Condurso et al., 2012). Grafting is a bridge between two genetically different parts that include rootstock and scion. Successful grafting is predominantly the result of complex histological, structural, and physiological changes at the graft junctions (Pina et al., 2017). Compatible combinations can establish graft union, callus tissue, and cambium formation between vascular bundles of both rootstock and scion. However, some rootstock and scion combinations cannot bridge together to form a successful graft union and this phenomenon is generally referred to as graft incompatibility in litchi (Chen et al., 2017) along with other fruits (Andrews and Marquez, 2010). In fruit plants, apart from total incompatible that is known graft failure. The translocated as well as localized are other two types of graft incompatibility which has been reported in fruits (Reig et al., 2018; Amri et al., 2021). The main reasons of graft failure include genetic distance, poor craftsmanship, environmental stresses and pathogens attack (Tedesco et al., 2020). Despite the significant application of grafting in horticultural crops, its genetic mechanism is still confusing although progress has been achieved in recent years.

The success of grafting is variable in litchi due to graft incompatibility between different rootstock and scion combinations (Chen et al., 2017). The ability to select suitable rootstock coupled with easy of wide-scale grafting application is therefore critical to increase fruit yield. Lack of graft compatible combinations is a main limiting factor for large-scale propagation of litchi. In this way, influence the profit return, performance, and longevity of commercial orchards. The physiological and biochemical aspects associated with grafting success were widely investigated in various fruit crops (Cookson et al., 2013; Xu et al., 2015; Dogra et al., 2018). The previous research data has revealed that carbohydrates (Moing et al., 1990), phytohormones (Nanda and Melnyk, 2018), phenolic acids (Canas et al., 2015), lignins, and flavonoids compounds (Pina and Errea, 2008; Reig et al., 2018) influence the mechanism of wound healing, tissue fusion, and vascular rejoining between scion and rootstock. In addition, anti-oxidant activities of superoxide, peroxidase, and superoxide dismutase contribute to different stages of graft union formation (Aloni et al., 2008). Because of recent advancements in biotechnology, the transcripts, proteins, and metabolites analyses have widely been applied to investigate the difference between compatible and incompatible graft combinations in various horticulture crops (Wang et al., 2016; Prodhomme et al., 2019; Bantis et al., 2021). Altered expression of *phenylalanine ammonia lyase (PAL)* genes had significant coloration with phenolic, flavonoids, and antioxidant enzymes activity in the scion grafted on the incompatible rootstock (Pereira et al., 2017). The *indole-3-acetic acid (IAA)* along with *auxin response factor (ARF)* encoding genes mediate various biological changes to promote the vascular connection between the scion and the rootstock (Yin et al., 2012). In addition, transcriptional reprogramming and epigenetic changes through histone deacetylation and acetylation regulate the process of wound healing at the graft interface (Kapazoglou et al., 2021). Overall, the phenotypic to genotypic association for incompatible combinations is challenging and it requires a large planting area, resources, and time to discover genetic control of this target trait.

Despite extensive utilization of the grafting propagation technique, the genetic mechanism underlying wounding healing, callus formation, cell expansion, and cell differentiation is not yet fully understood in most grafted plants (Gaut et al., 2019). In particular, limited information is available in litchi about the key genes, pathways, and metabolites that regulate the causes of graft incompatibility. Recently, integrated transcriptomic and metabolomics data provide a platform to explore the molecular mechanism of the target trait (Shen et al., 2021). Metabolomics analysis provides an effective way to determine the composition and concentration of metabolite compounds. In addition, transcriptomic data is particularly important to identify network linkage among key genes and metabolites. In litchi, previous research revealed that most rootstock cultivars of Heli (also known Huaizhi) had good compatibility with various scions whereas most rootstock cultivars of Heiye (also known Hak Yip) showed poor compatibility with various scions (Galan et al., 2018). Thus, to minimize this research gap on graft incompatibility in litchi, metabolomics and transcriptomic analyses were performed by using compatible and incompatible graft combinations in this study. Our results identified key metabolic components, characterized their gene expression, and established a preliminary genetic mechanism of graft incompatibility in litchi. This study provides an important platform to identify candidate genes and metabolic substances pertinent to graft incompatibility in litchi.

## 2 Results

### 2.1 Graft-incompatibility test experimentation among two litchi varieties

**FIGURE 1:**
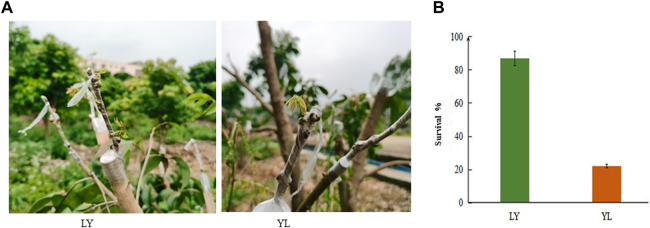
Different grafting combinations of litchi with survival rate in the field **(A)** LY shows Heli rootstock with Heiye scion graft combination whereas YL shows Heiye rootstock with Heli scion graft combination **(B)** the percentage of graft survival in LY and YL graft combinations of litchi.

The joining of rootstock and scion sometime leads to complete failure of grafting in litchi and this phenomenon is often called graft-incompatibility. This study utilized two different litchi varieties to examine graft-incompatibility. Heli is a broad compatibility rootstock variety used in China whereas Heiye is a weaker rootstock variety. During mutual grafting experimentation, Heli was used as the rootstock and Heiye as a scion (LY) along with Heiye was used as the rootstock and Heli as a scion (YL) (Figure 1A). It was observed that LY showed graft compatibility among rootstock and scion. In contrast, YL exhibited graft incompatibility. The investigation of field grafting (20 replicates of each combination) 1 month later showed a significant difference in the survival rate of grafting between LY and YL. The grafting survival rate was greater in LY than in YL (Figure 1B). This lower survival rate in YL is possibly associated with biochemical and molecular changes at graft union interference.

### 2.2 Metabolomics analysis

**FIGURE 2:**
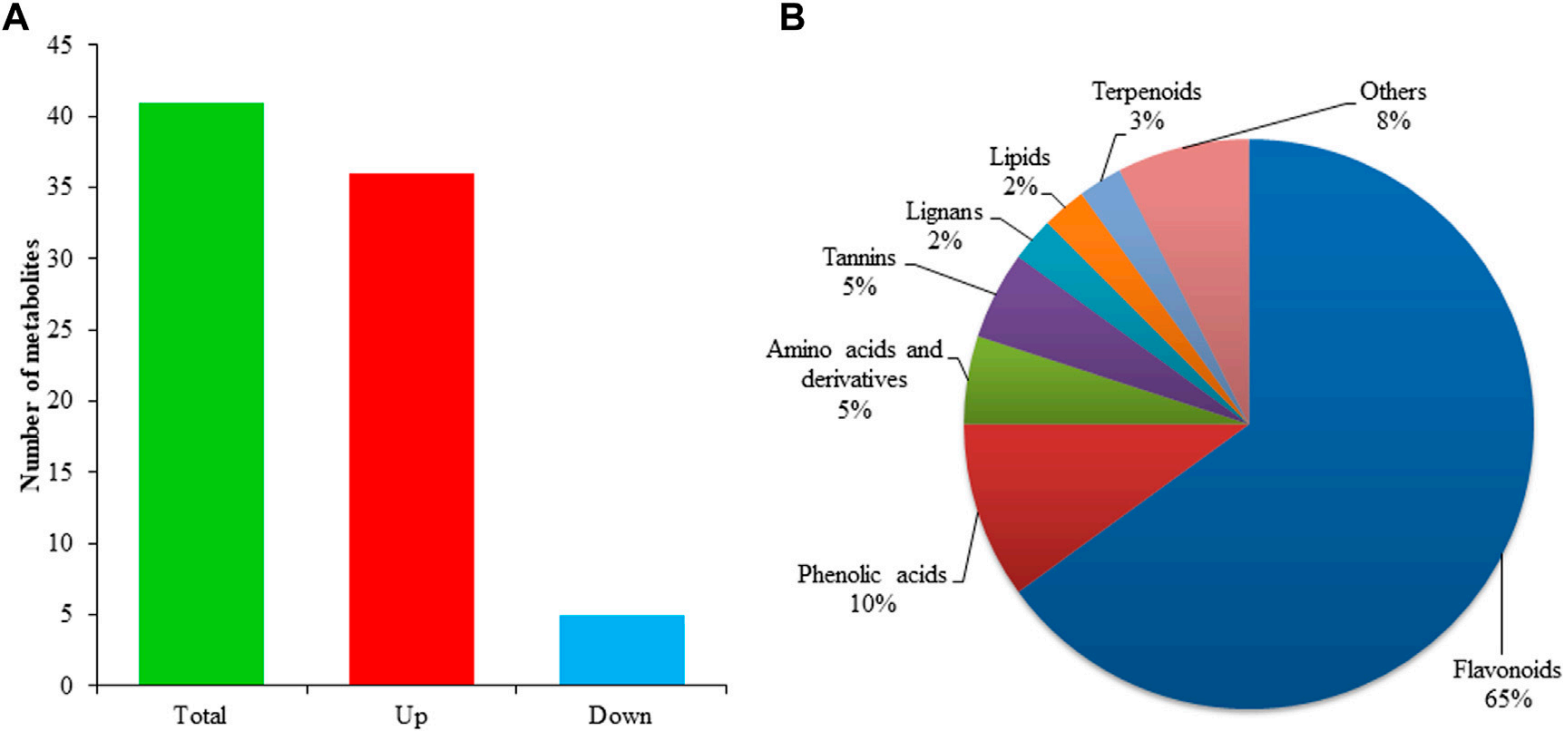
Statistics of differential metabolites and their classification in percentage **(A)** distribution of differentially accumulated metabolites identified in LYvsYL graft combinations of litchi **(B)** classification of differentially accumulated metabolites.

The metabolomics analysis is vital to understanding the composition and concentration of metabolic substances for target traits. Thus, ultra-performance liquid chromatography-mass spectrometry was used to investigate the abundance of metabolites in both grafting combinations of litchi. A total of 1,166 metabolites were detected in grafted litchi samples (Supplementary Table S1). The majority of detected metabolites belonged to different subclasses of phenolic acids, amino acids, and organic acids followed by flavonoids, lignins, alcohols, and free fatty acids. The comparative analysis revealed that 41 metabolites had shown a significant difference between graft compatible and incompatible combinations of litchi. Among all differentially accumulated metabolites, 36 metabolites showed higher accumulation in YL than LY whereas only five showed lower accumulation (Figure 2A). Most differentially accumulated metabolites belong to flavonoids followed by phenolic acids, amino acids, others, and lignin (Figure 2B).

**FIGURE 3:**
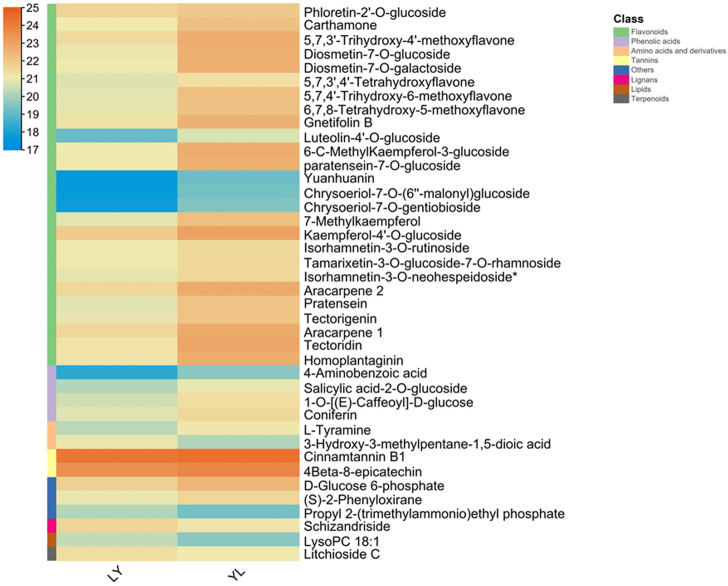
The abundance of differential metabolites in LY and YL graft combinations of litchi.

**FIGURE 4:**
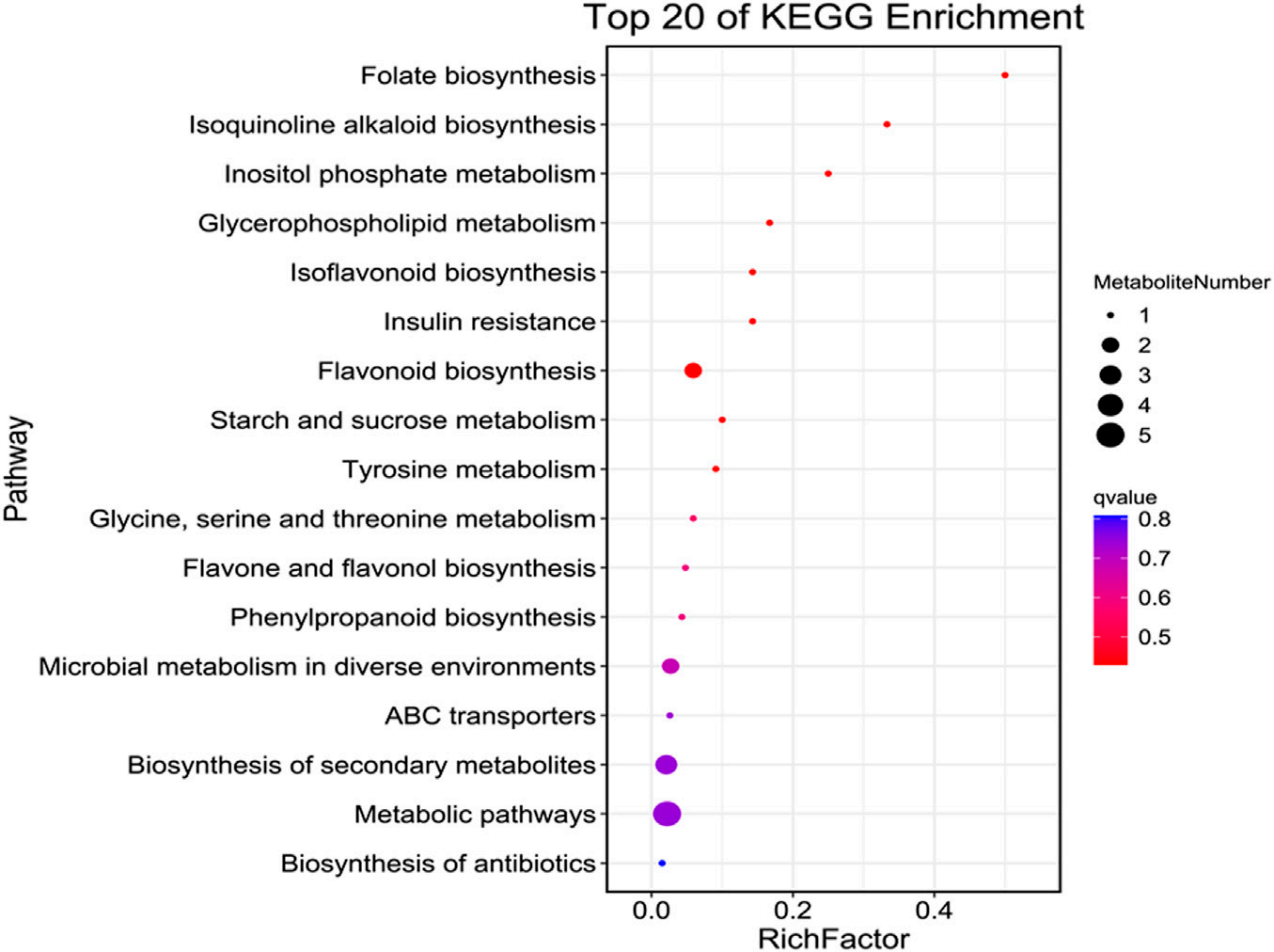
Pathway enrichment of differential metabolites identified LYvsYL graft combinations of litchi.

In addition, all metabolites had shown dynamic variations between LY and YL graft combinations of litchi. In particular, flavonoids compounds such as 5,7,3'-trihydroxy-4'-methoxyflavone, 5,7,4'-trihydroxy-6-methoxyflavone, and 7-methylkaempferol along with other subclasses had significantly higher abundance in graft incompatible combination than compatible. Additionally, phenolic acid compound salicylic acid-2-O-glucoside and coniferin showed similar abundance in this combination (Figure 3). However, lignin compound schizandriside exhibited many fold lower abundance in graft incompatible combinations than compatible. Pathway enrichment analysis revealed the majority of differential metabolites exhibited functional annotation with the biosynthesis of flavonoids and isoflavonoids (Figure 4). Overall, graft incompatibility generates various biochemical changes in litchi. Especially, the level of flavonoids and phenolic acids along with lignin changes significantly in response to graft incompatibility in litchi.

### 2.3 Transcriptome analysis

**FIGURE 5:**
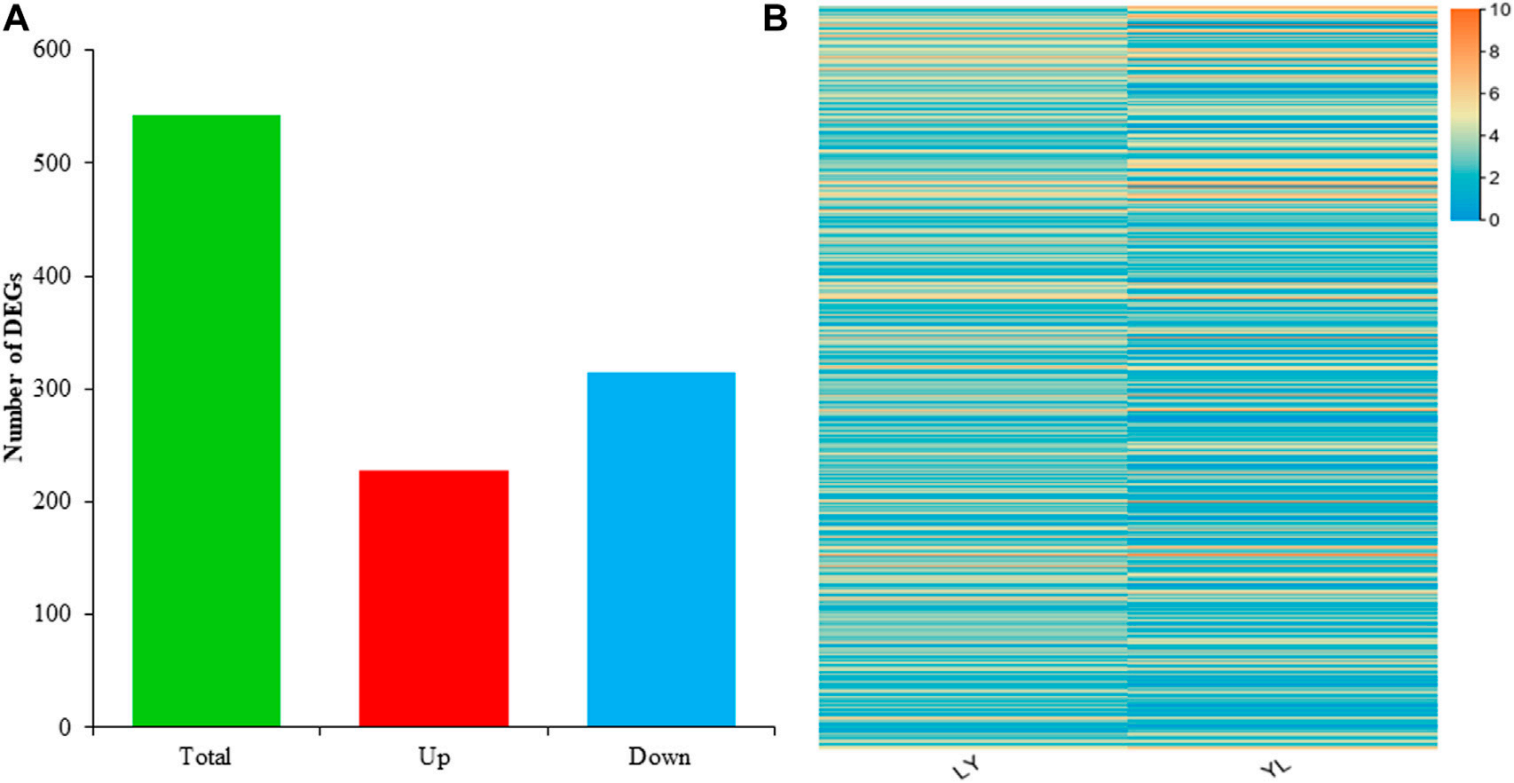
Distribution and expression of differential genes identified in LYvsYL graft combinations of litchi **(A)** distribution of differential genes **(B)** heatmap of differential expressed genes.

To investigate the transcripts associated with grafting incompatibility in litchi, RNA sequencing was performed using grafted samples harvested from graft compatible LY and incompatible YL combinations. The overall sequence mean of raw reads was 7038200400 for LY and 7182378150 for YL (Supplementary Table S2). The mean of valid reads in the LY was 6973474423 with Q30% of 92.3. In contrast, the mean of valid reads in the YL was 7116452465 with a Q30% value of 92.1. It was observed that almost 83% of reads were mapped to the reference genome of litchi. Of which, 79% were uniquely mapped and 4.4% were multi-mapped reads in each library. These findings revealed high quality and a large quantity of sequenced data. In addition, it confirms the precision of assembly and coverage of the transcripts in this study. The expression of genes was calculated as fragments per kilobase of exon model per million reads mapped (FPKM) to understand dynamic changes of transcriptomes between compatible and incompatible grafted litchi plants. A total of ∼32500 genes were expressed in each dataset. The differentially expressed genes (DEGs) between two grafted samples were selected at *p* ≤ 0.05 and with log2 (fold change) > 1 or < −1. The comparative analysis of LYvsYL showed a total number of 542 DEGs. The distribution analysis determined 314 down-regulated and 228 upregulated genes in YL as compared to LY (Figure 5A). All these DEGs had distinct mean expressions among both graft combinations of litchi with the range from 0 to 10 fold change (Figure 5B). The overall comparative analysis of DEGs revealed that grafting most likely caused dynamic changes in genes expression and a large number of genes regulate graft incompatibility in litchi.

### 2.4 Functional analysis of the DEGs

**FIGURE 6:**
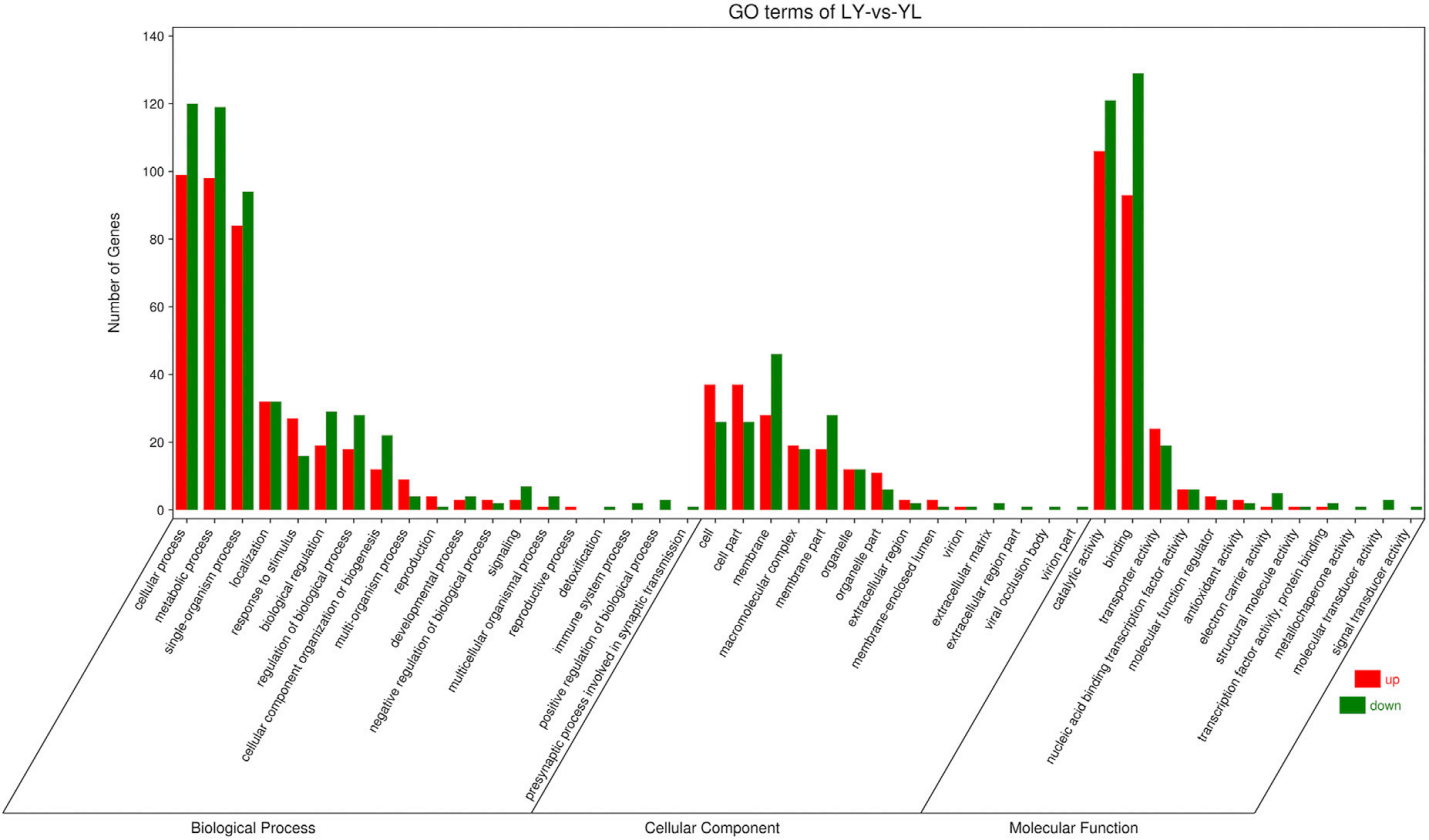
Functional enrichment for differential genes identified in LYvsYL graft combinations of litchi.

**FIGURE 7:**
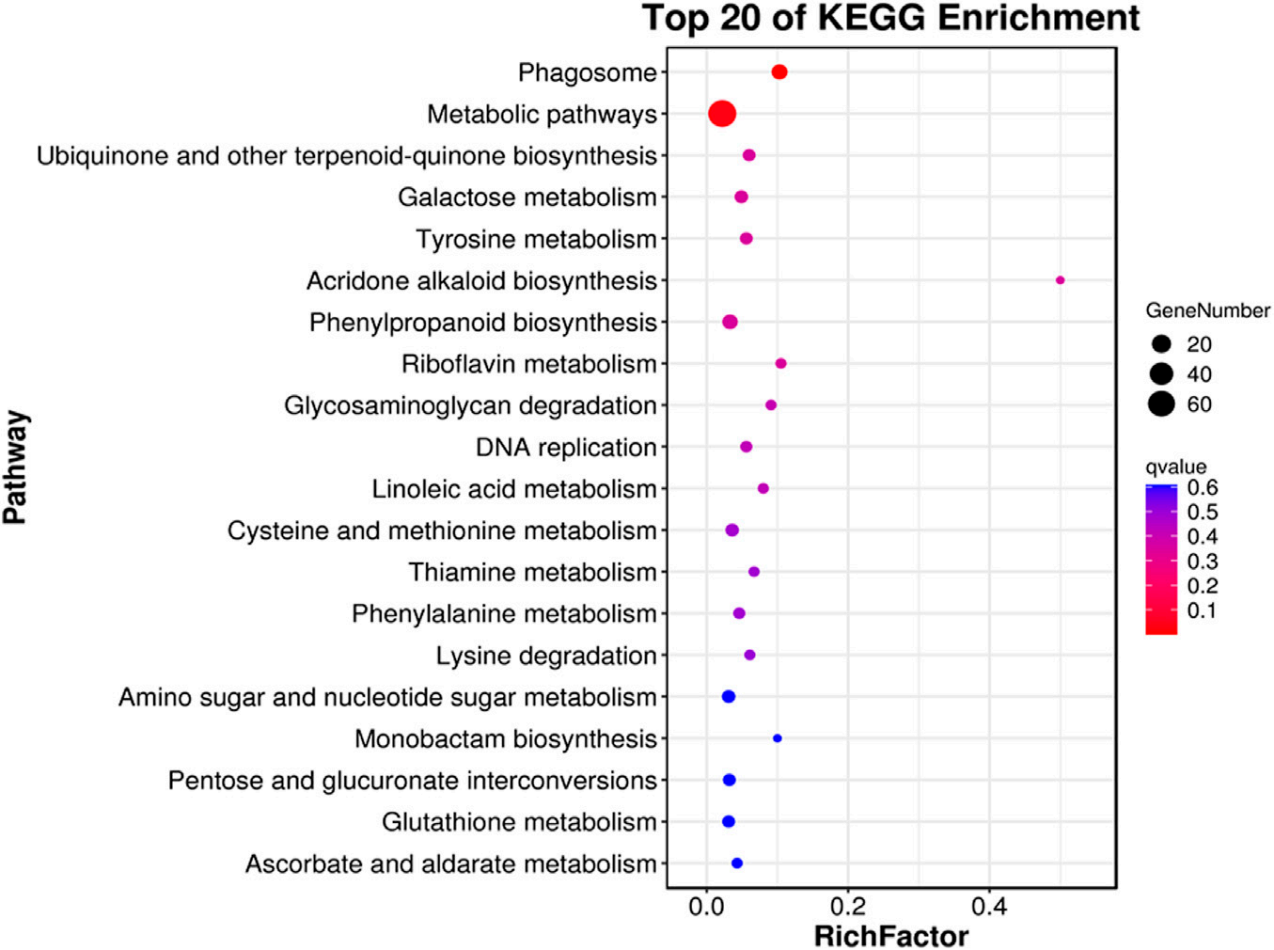
Pathway enrichment for differential genes identified in LYvsYL graft combinations of litchi.

The majority of the DEGs performed biological functions interlinked with cellular and metabolic processes while cellular functions related to cell and membrane components. In addition, most DEGs influence the catalytic and binding activities at the molecular level (Figure 6). Pathway enrichment revealed that most DEGs had shown enrichment in basic pathways related to biosynthesis of various metabolic compounds. In addition, the phagosome pathway followed by the phenylpropanoid biosynthesis had more significance and enriched genes (Figure 7). Functional analysis of the DEGs indicates that rootstock and scion joining disturbs the phagosome as well as phenylpropanoid biosynthesis pathway encoding genes which ultimately influence the mechanism of grafting incompatibility in litchi.

#### 2.4.1 Phenylpropanoid biosynthesis pathway disturbance during grafting in litchi

**FIGURE 8:**
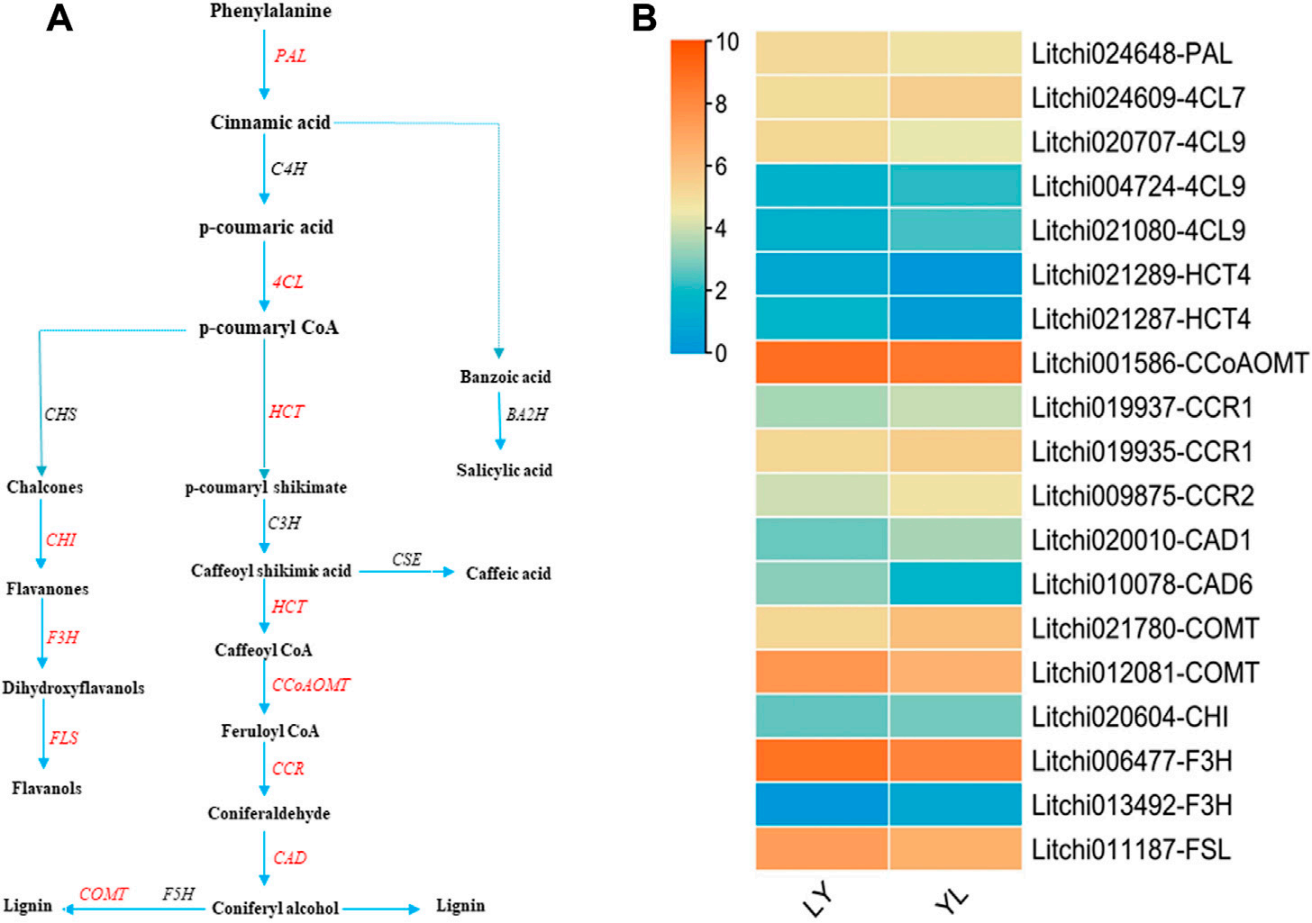
Regulation of phenylpropanoids pathways structural genes identified in LYvsYL graft combinations of litchi **(A)** general diagram of biosynthetic pathways of phenylpropanoids including flavonoids, monolignols, and phenolic acids. PAL: phenylalanine ammonia lyase, C4H: cinnamate 4-hydroxylase; 4CL, 4-coumaroyl CoA ligase, CHS: chalcone synthase, CHI: chalcone isomerase, F3H: flavanone -3-hydroxylase, FSL: flavonol synthase, HCT: hydroxycinnamoyl-CoA shikimate/quinate hydroxycinnamoyl transferase, CEH: p-coumarate 3-hydroxylase, CCoAOMT: caffeoyl-CoA O-methyl transferase, CCR: cinnamoyl-CoA reductase, CAD: cinnamyl alcohol dehydrogenase, F5H: ferulate 5-hydroxylase, COMT: caffeic acid O-methyltransferase, BA2H: benzoic acid 2-hydroxylase, and CSE: caffeoyl shikimate esterase. Red text genes showed differential regulation in this study **(B)** expression heatmap of differentially expressed genes in LY and YL graft combinations of litchi.

Phenylpropanoids regulate all phases of plant responses against various stress stimuli by generating an array of secondary metabolites derived from phenylalanine. In plants, the phenylpropanoid pathway mediates the biosynthesis of monolignols, flavonoids, and phenolic acids (Vogt, 2010; Deng and Lu, 2017). In brief, the phenylalanine is first converted into p-coumaryl CoA *via* different enzymes that include phenylalanine ammonia lyase (PAL), cinnamate-4-hydroxylase (C4H), and 4-coumaroyl coenzyme A ligase (4CL). The p-coumaryl CoA then produces basic precursors for the biosynthesis of lateral metabolites (Figure 8A). Interestingly, several genes involved in the biosynthesis of lignin, flavonoids, and phenolic acids had shown dynamic expression changes between graft compatible and incompatible combinations of litchi. For instance, *Litchi024648-PAL* had a significant higher expression level in LY than YL graft combination. In contrast, *Litchi021080-4CL, Litchi004724-4CL9,* and *Litchi021080-4CL9* exhibited up-regulation in YL (Figure 8B).

Lignin along with cellulose is the most abundant polymer, synthesizes from monolignols in the phenylpropanoid pathway, and induces stability in response to external damages like wounds in plants (Rinaldi et al., 2016). The different kinds of lignin are formed *via* various enzymes that include hydroxycinnamoyl-CoA shikimate/quinate hydroxycinnamoyl transferase (HCT), p-coumarate 3-hydroxylase (C3H), caffeoyl-CoA O-methyl transferase (CCoAOMT), cinnamoyl-CoA reductase (CCR), cinnamyl alcohol dehydrogenase (CAD), ferulate 5-hydroxylase (F5H), and caffeic acid O-methyltransferase (COMT) (Figure 8A). A total of ten genes encoding enzymes correlated with lignin biosynthesis were identified in this study. All these genes showed significant dynamic expression differences between graft compatible and incompatible combinations of litchi. In particular, the expression of *Litchi021287-HCT4, Litchi021289-HCT4, Litchi001586-CCoAOMT*, *Litchi010078-CAD6,* and *Litchi012081-COMT* exhibited up-regulation in LY. However, *Litchi019935-CCR1, Litchi019937-CCR1, Litchi009875-CCR2, Litchi020010-CAD1,* and *Litchi021780-COMT* had up-regulation in YL (Figure 8B). Their altered expression most likely disrupts lignin formation at graft union in response to wounding and may play a key role in graft in-compatibility in litchi. In addition, four genes annotated as *Litchi020604-CHI, Litchi006477-F3H, Litchi013492-F3H*, and *Litchi011187-FSL*, and involves in the biosynthesis of flavonoids showed significant differences in response to grafting (Figure 8B). This might be the main reason that majority of metabolites detected in both graft combinations belong to the flavonoids group. In plants, the flavonoids participate in various biological functions, regulate antioxidant defense mechanisms in response to various stresses, and affect cell growth and differentiation (Agati et al., 2012). So, an imbalance of flavonoid metabolic substances at graft junction may result in physiological and biochemical disorders that induce graft incompatibility. In brief, phenylpropanoid pathway disturbance plays an imperative role in graft incompatibility in litchi.

#### 2.4.2 Phagosome pathway disturbance during grafting in litchi

**FIGURE 9:**
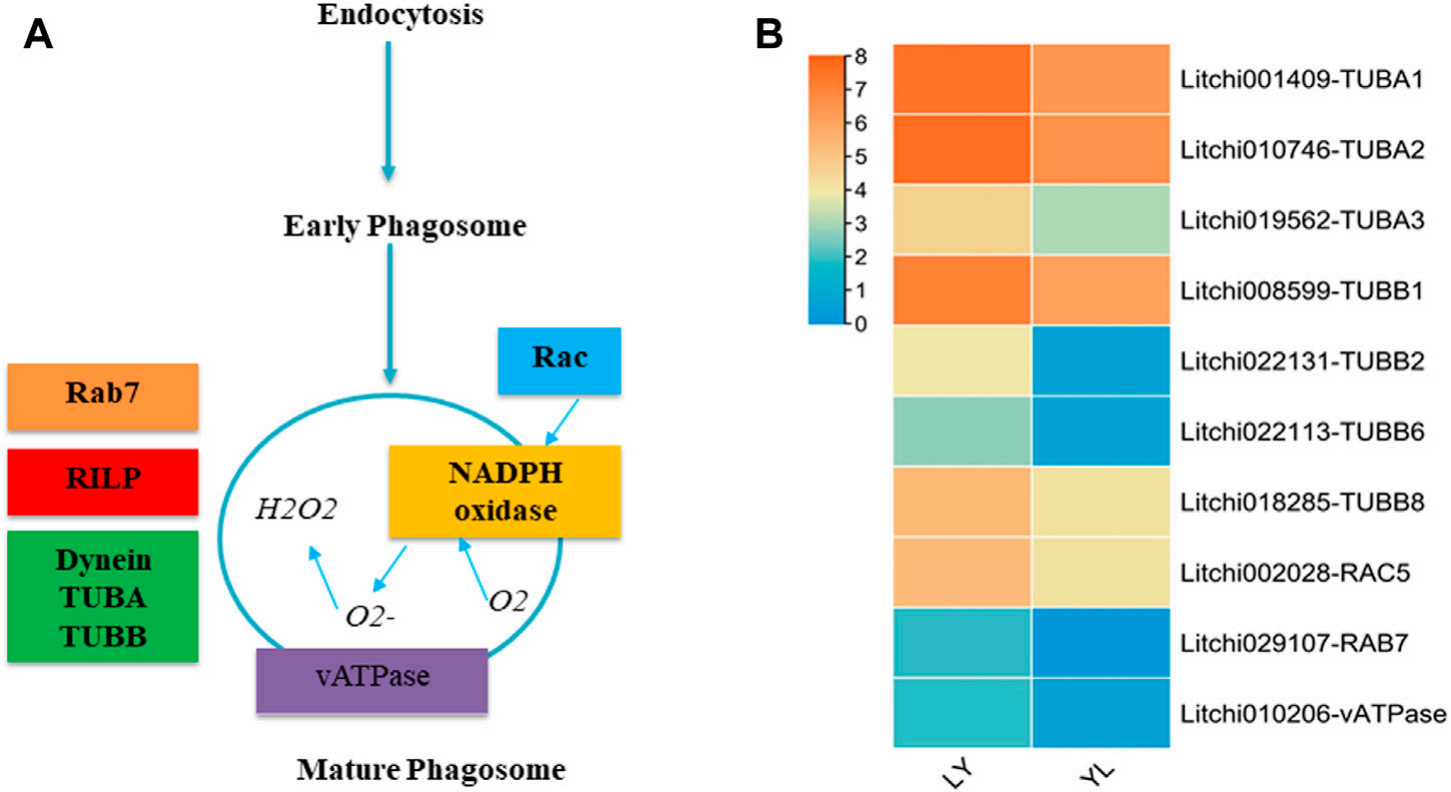
Regulation of the phagosomes pathway genes identified in LYvsYL graft combinations of litchi. **(A)** The simplest diagram of the mature phagosome. TUBA: tubulin alpha chain, TUBB: tubulin beta chain, RAC: rac-like GTP-binding protein, RAB: rab-like GTP-binding protein, vATPase: V-type proton ATPase subunit a3-like, and RILP: Rab interacting lysosomal protein **(B)** expression heatmap of differentially expressed genes in LY and YL graft combinations of litchi.

The phagosomes play an essential role to regulate the process of phagocytosis. Phagocytosis maintains cellular homeostasis by removing cell debris and responding to invading organisms (Lee et al., 2020). The matured phagosomes are formed with the interaction to other cell organelles *via* complex chemical reactions regulated by different enzyme encoding genes (Figure 9A). In our target analysis, nine genes enriched in the phagosome pathway had shown significant differential expression between graft compatible and incompatible combinations of litchi. Especially, tubulin alpha chain annotated genes *Litchi001409-TUBA1*, *Litchi010746-TUBA2, and Litchi019562-TUBA3* exhibited many fold higher expression in LY than that in YL graft combination of litchi (Figure 9B). The tubulin beta chain related genes that include *Litchi008599-TUBB1*, *Litchi022131-TUBB2, Litchi022113-TUBB6*, and *Litchi018285-TUBB8* were up-regulated expression in LY. In addition, vATPase, Rab, and Rac-like protein encoding genes had shown higher regulation in LY than YL graft combination of litchi. The alpha and beta tubulin are major components of microtubules in plants. Apart from their specific physiological role, microtubules influenced the separation of the chromosome during cell division and the cell elongation process. Besides, they are predicted to regulate the development of secondary cell walls in plants (Radchuk, 2008). The downregulation of several tubulin genes in YL might lead to an imbalance in cell division and cell wall composition in response to wounding. In this way, may induce graft incompatibility in litchi.

### 2.5 Association analysis between transcripts and metabolites

**FIGURE 10:**
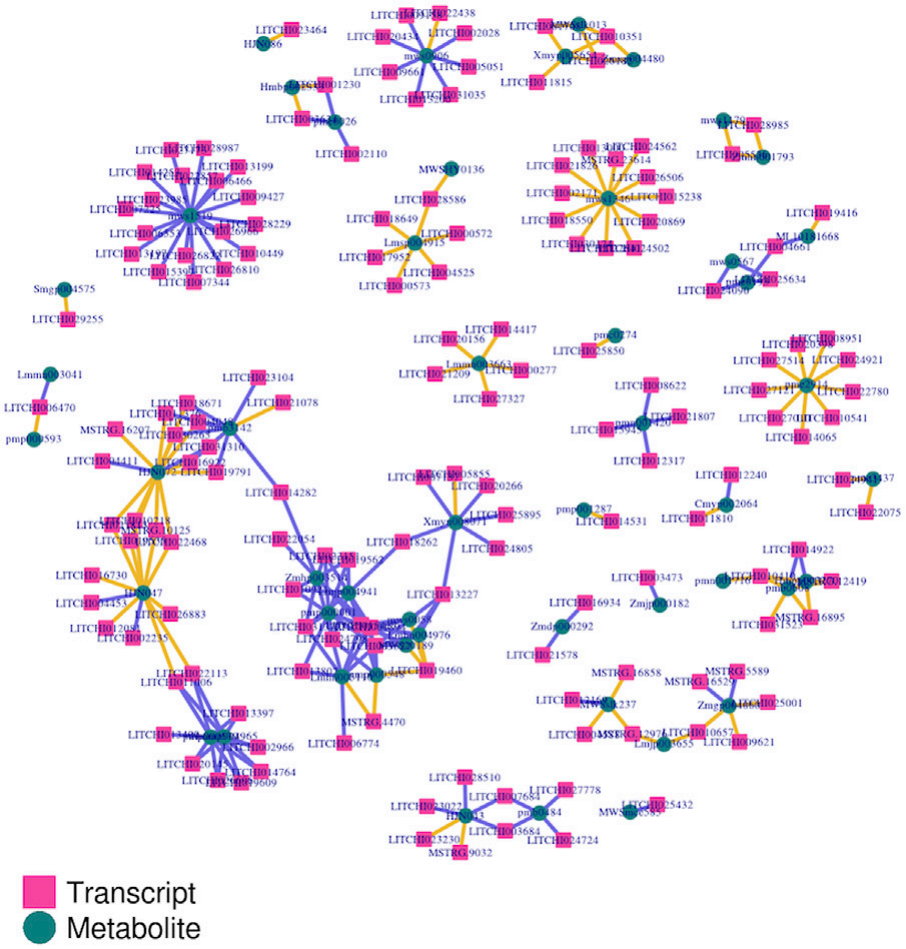
Overview of network linkage between differential genes and differential metabolites identified in LY and YL graft combinations of litchi.

The transcripts and metabolites integration is vital to understanding the functional mechanism of the target traits. Based on all transcriptomic and all metabolomic data, we performed two-way orthogonal PLS analysis. This model is suitable for association analysis of two data components, enabling bidirectional modeling, and prediction in two data matrices. It explained the total variation of each part with the explanatory power of R2. The analysis stated that 189 genes had significant strong association with 132 metabolites. The association value was either strongly positive or negative with a range of above 0.98 or -0.98 (Supplementary Table S3). However, most metabolites had shown a significant negative association with genes. In particular, flavonoids such as pratensein, 5,7,4'-trihydroxy-6-methoxyflavone, aracarpene-1, and tectorigenin exhibited a significant negative association with many genes. The phenolic acids including salicylic acid-2-O-glucoside and coniferin had a similar relationship with many genes. In addition, schizandriside belonging to the class of lignin were other metabolites that showed a significant negative association with different genes. Only genes and metabolites which showed strong association were screened to execute the final transcript-metabolites network analysis. In the final network, the lignin pathway encoding gene *Litchi012081-COMT* displayed network connection with lignin metabolite schizandriside. Besides this, the phagosome pathway gene *Litchi022113-TUBB6* had network linkage with metabolites including lignin compound schizandriside and flavanone compound diosmetin-7-O-glucoside (Figure 10). However, *Litchi008599-TUBB1* linkage was comprised of flavonol compound 7-methylkaempferol along with two flavones compounds 5,7,4'-trihydroxy-6-methoxyflavone and 6,7,8-tetrahydroxy-5-methoxyflavone. *Litchi002028-RAC5* had shown network linkage only with phenolic acid compound coniferin. Furthermore, our integrated analysis identified that flavonoid compounds 5,7,4'-trihydroxy-6-methoxyflavone and 6,7,8-tetrahydroxy-5-methoxyflavone had strong network linkage with several genes. These can be therefore regarded as hub metabolites that influenced graft compatibility or in-compatibility in litchi. In brief, disruption in flavonoids and lignin biosynthesis along with their tightly linked genes possibly influences the biochemical and molecular processes associated with the healing, tissue fusion, and vascular reconnection in grafted litchi plants. Further in-depth research can be helpful to explore how these genes and metabolic interaction lead to graft-incompatibility in litchi.

## 3 Discussion

Litchi is considered an economically important horticulture crop, specifically in China, and grafting has already proved the most efficient propagation measure to improve specific traits of interest in the orchards. As expected, several rootstock and scion combinations cannot generate compatible grafting in litchi. Thus, graft incompatibility is a challenge and a major constraint in large-scale profitability of commercial orchards. Accordingly, this study analyzed the metabolites and gene differences among graft compatible or incompatible combinations. Furthermore, discussed how metabolites and their regulatory genes mediate different aspects of graft incompatibility in litchi.

### 3.1 Abundance of polyphenols regulate graft incompatibility in litchi

Graft incompatibility is a complex biological disorder produced by the biochemical and physiological interactions between scion and rootstock. In plants, complex biological disorders are frequently related to the abundance of individual metabolites or the interaction of specific metabolites (Fiehn et al., 2000). Our study determined that grafting generates a diversity of metabolic compounds in litchi. The phenolic acids followed by flavonoids, lignins, alcohols, and free fatty acids were the most abundant than other metabolites. In grafted plants, the production and stimulation of specific metabolites disturb the formation of graft union, callus, and vascular cells through an imbalance in biochemical pathways. Many phytochemical analyses have shown polyphenols especially phenolic acids, flavonoids, and lignin affect different phases of scion and rootstock interaction (Canas et al., 2015; Assunção et al., 2016). In various graft incompatible combinations of fruit crops, higher accumulation of gallic acid, gentisic acid, ellagic acid, p-coumaric acid was detected at graft junction (Cooman et al., 1996; Usenik and Stampar, 2001). This abundance of polyphenols ultimately causes dysfunction in process of development, cell division, and differentiation in response to wounding. In addition, they have an important effect on auxin levels and therefore may disturb the development of vascular cells in graft incompatible scion and rootstock combinations (Aloni et al., 2010). In this study, phenolic compounds such as 4-aminobenzoic acid, salicylic acid-2-O-glucoside, 1-O-(E)-caffeoyl-D-glucose and coniferin showed significantly higher accumulation in graft incompatible combinations than compatible. These phenolics most likely affect the mechanisms of graft failure in litchi by reprograming various biochemical components. It has been shown that phenolic substances transported from vacuole to cytoplasm induced inhibition of the lignification process in less compatible graft combinations (Reig et al., 2019). Lignin in addition to cellulose is a key component of xylem vessels and plays a crucial role in transportation of nutrients. Their imbalance can produce a week connection between scion and rootstock. Interestingly, lignin compounds schizandriside was significantly lower in graft incompatible combinations of litchi. The flavonoid components involved in antioxidant defense mechanism are predicted to be critical for graft union formation (Assunção et al., 2016). At earlier stages of grating, high oxidative stress prevails in response to wounding which leads to abnormal physiological functions as well as damage to cell structure. The plant in response probably activates the accumulation of flavonoids and other antioxidant enzymes at graft junction (Assunção et al., 2019). Previous studies have shown that the activity of antioxidant agents at graft union was frequently higher in graft incompatible combinations than compatibles (Nocito et al., 2010). Similarly, the accumulation of flavonoids has already been stated in incompatible graft combinations of various fruit crops (Hudina et al., 2014; Irisarri et al., 2015). Our metabolic analysis found that various species of flavanones, flavones, flavonols, chalcones, and isoflavones had a higher level in graft incompatible combinations than compatible. Consequently, the composition and concentration of polyphenols can be important indicators of graft incompatibility in other fruit plants (Prabpree et al., 2018) and litchi has no exception. In addition, combinations of metabolites rather than the existence of individual substances have more biological relevance to the complex mechanism of graft incompatibility in litchi.

### 3.2 Insights into the regulatory mechanism of graft incompatibility in litchi

The genetic mechanism underlying graft incompatibility is yet poorly understood in litchi. This knowledge is essential to improve the efficiency of screening and release of new rootstocks or scions. To understand the genetic constituent of graft incompatibility in litchi, transcriptome comparison was performed to determine the profiles of differentially expressed genes among compatible and incompatible graft combinations. Our result observed that few quantitative differences in gene expression probably regulate unsuccessful graft phenotype in litchi. In addition, most of these changes showed function annotation with metabolic components, especially in the phenylpropanoids pathway. The phenylpropanoids are a large group of secondary metabolites that contains a diversity of flavonoids, monolignols, and phenolic acids. Almost, all phenylpropanoid pathway genes have been characterized in plants (Noel et al., 2005; Vogt, 2010). In the initial phase of this pathway, phenylalanine produces the p-coumaryl CoA through chemical reactions catalyzed by *PAL*, *C4H*, and *4CL* genes. These genes are compulsory for the biosynthesis of all subsequent species of polyphenols. In addition, their expression is changed in response to the growth stage and stress (Hamberger et al., 2007). In this study, the expression level of *Litchi024648-PAL* was significantly different among graft incompatible and compatible combinations of litchi. According to previous research, differential regulation of *PAL* genes plays a crucial role to generate graft incompatibility signs in fruit crops (Irisarri et al., 2015; Chen et al., 2017). Moreover, these genes help to generate phenolic compounds, and therefore affect the callus development during the early phase of scion and rootstock connection (Pina and Errea, 2008). Our result further identified that *Litchi021080-4CL, Litchi004724-4CL9, and Litchi021080-4CL9* had significant up-regulated expression in graft incompatible combinations. Their up-regulation possibly produced higher levels of phenolic acids especially 4-aminobenzoic acid, salicylic acid-2-O-glucoside, and coniferin. Finally, the abundance of these phenolic substances might cause growth dysfunction and create the phenotypic expression of graft incompatibility in litchi. Functional genomic research can help to explore the relationship between phenolic substances and graft incompatibility of litchi.

In woody plants, lignin is polymerized from three monolignols that include the p-coumaryl, coniferyl, and sinapyl alcohols. Besides other functions, lignin through the lignification process facilitates cell wall stability in response to wounding in plants (Liu et al., 2018). The monolignols, the building block of lignin are primarily synthesized from phenylpropanoid units through a series of chemical reactions catalyzed by various enzymes encoding genes such as *HCT, CCR, CAD,C3H/C4H, COMT*, and *CCoAOMT* (Davin et al., 2008; Yoon et al., 2015). In our results, the expression of *Litchi021287-HCT4, Litchi001586-CCoAOMT, Litchi010078-CAD6, and Litchi012081-COMT* were many fold lower in graft incompatibility combination of litchi. In addition, the abundance level of lignin compound schizandriside was significantly lower in this combination. Remarkably, our network analysis also confirmed a strong linkage between *Litchi012081-COMT* and schizandriside. These results predict that the lower level of lignin substances might decrease graft wound healing which results in a week connection between scion and rootstock in graft incompatibility combination of litchi. In addition to phenolic acids and lignin biosynthesis, flavonoids are also synthesized from branches of the phenylpropanoid pathway *via CHS, CHI, FEH,* and *FSL* enzyme annotated genes in plants (Aoki et al., 2000). This study observed that *Litchi020604-CHI, Litchi006477-F3H, Litchi013492-F3H, and Litchi011187-FSL* displayed dynamic expression changes in response to graft incompatibility*.* These expressions most likely altered the profiling of flavonoids in graft incompatible combinations of litchi. It is well known that flavonoids are powerful antioxidant agents in plants. In particular, flavanols along with peroxidases reduced oxidative damage (Agati et al., 2012; Sharma et al., 2012). As high oxidative stress is prevails in response to graft incompatibility in woody plants, and this ultimately cause higher abundance of flavonoids in the incompatible graft combinations of various fruit crops (Musacchi et al., 2000; Hudina et al., 2014; Amri et al., 2021). Therefore, our results presumed that the abundance of flavonoids and altered expression of their regulatory genes induce graft incompatibility in litchi. However, an in-depth research is mandatory to clear how phenylpropanoid pathway disturbance mediates graft incompatibility in litchi. Besides expression differences in structural genes of phenylpropanoids, several tubulin family genes exhibited down-regulation in incompatible graft combinations of litchi. Our integrated transcriptomic and metabolomics further identified that the tubulin genes had strong network linkage with many flavonoid compounds. Being the core elements of microtubules, down-regulation of tubulin genes might disrupt the mechanism of mitosis, mechanical stress, intracellular transport, cell division, and cell morphogenesis (Meiring et al., 2020) in graft incompatible combinations of litchi. In concise, our study identified an array of potential genes and metabolites that are tightly correlated with graft incompatibility of litchi. However, functional studies integrated with advanced genome editing tools could be a step further to elucidating the genetic mechanism of graft incompatibility of litchi.

## 4 Conclusion

This study highlights the changes in affinity caused by the exchange of rootstock and scion positions in litchi. Metabolic data analysis revealed abundance levels of various methoxyflavones significantly increased in response to incompatible scion and rootstock interaction. In contrast, lignin compound schizandriside exhibited many fold lower abundance in graft incompatible combinations. Transcriptomic analysis identified that dynamic changes in flavonoids, phenolic acids, and lignin pathway genes play an important role in survival rate of grafting. In particular, *Litchi024648-PAL, Litchi021080-4CL9, Litchi013492-F3H, Litchi011187-FSL, Litchi021287-HCT4, Litchi010078-CAD6, Litchi019935-CCR1, Litchi012081-COMT*, and *Litchi008599-TUBB1* are predicted to induce graft incompatibility in litchi. The integration of metabolic and transcriptomic data further confirmed a strong linkage between potential genes and metabolic substances that probably facilitate graft incompatibility. Our results provide useful insights into the biochemical and genetic aspects of graft incompatibility in litchi.

## 5 Materials and methods

### 5.1 Plant materials and analysis of metabolic compounds

The plant materials utilized in our study comprised two different litchi varieties that include Heli (known as Huaizhi in Guangdong) and Heiye. In China, Heli is a broad compatibility rootstock variety. In contrast, the Heiye is a weaker rootstock variety as compared to Heli. For mutual grafting, Heiye was utilized as a scion and grafted on the rootstock of Heli (LY). Then, Heli was used as a scion and grafted on Heiye (YL) rootstock. The survival rates of successful grafting were measured after 1 month from 100 plants for each graft combination. The grafted samples were harvested in triplicates from the healing site for graft compatible (LY) and incompatible (YL) combinations of litchi, frozen instantly in liquid nitrogen and stored in an ultra-low temperature refrigerator at −80°C before further analyses. To study the metabolic profiles, the biological samples were converted into powder form with a grinder (MM 400, Retsch) at a 30 Hz frequency for 2 minutes. The 100 mg of the powder was mixed into 1.0 ml of 70% methanol containing 0.1 mg/L lidocaine as an internal standard and stored at 4°C overnight to perform the extraction. The overnight stored samples were vortex three times and centrifugation was then performed at 10,000 g for 10 min to produce more sufficient extraction. The supernatant liquid was aspirated, filtered through a microporous membrane (0.22 μm pore size), and finally transferred in injection vials for subsequent metabolomics analysis. In this study, the metabolic datasets were obtained from ultra-performance liquid chromatography (UPLC) (Shimadzu Nexera X2) integrated with tandem mass spectrometry (MS/MS) (Applied Biosystems 4500 QTRAP). The protocol of metabolic detection and their data generation was followed as briefly described in the previous study (Chen et al., 2013). The raw generated datasets were first filtered to obtain retention time, mass to charge ratio and peak intensity data matrix. The qualitative profiles of metabolites were analyzed in reference to MassBank, KNAPSAcK, HMDB, MoTo DB, METLIN along with other public databases (Wishart et al., 2012; Zhu et al., 2013). The orthogonal least partial squares discriminant analysis (OPLS-DA) combined with the student’s *t*-test was used to identify differential metabolites. The threshold values of VIP ≥1 and *p* ≤ *0.05* were mainly applied to identify significant differences between graft compatible and incompatible combinations of litchi (Saccenti et al., 2014).

### 5.2 RNA sequencing and transcripts data analysis

Total RNA was extracted from graft compatible and incompatible combinations by using the CTAB method (Gambino et al., 2008). Then, genomic DNA contamination was removed with the RNase-free DNase I (TaKaRa, Kyoto, Japan) kit to obtain high quality RNA. In addition, the presence of genomic DNA contamination was further confirmed with agarose gel electrophoresis. Accurate detection of RNA purity was performed with a Nanodrop spectrophotometer (Thermo Fisher Scientific, United States). The Qubit2.0 fluorescence meter was used to precisely measure the RNA concentration. Finally, the RNA integrity was checked on Bioanalyzer Agilent 2100 (Agilent Technologies, United States). To ensure the quality of sequencing, mRNA-Seq sample preparation kit was used to construct six sequenced cDNA libraries with standard quality. The libraries were then sequenced on the Illumina HiSeq2000 sequencing platform by Genedenovo Biotechnology Co., Ltd. (Guangzhou, China). The generated sequenced data was first filtered with the Fastp software to remove adapters, ambiguous bases, and low quality reads (Chen et al., 2018). The clean reads were aligned to the reference sequence of litchi (non-redundancy genome) with HISAT2 (Kim et al., 2015). The Stringtie was effectively applied to assemble transcripts (Pertea et al., 2015). The expression levels of all transcripts were determined using RSEM (Li and Dewey, 2011). The DESeq software was applied to identify differential genes between graft compatible and incompatible combinations of litchi (Love et al., 2014). Gene functional and pathway enrichment analyses were performed with Goatools (Klopfenstein et al., 2018) and KOBAS 2.0 (Xie et al., 2011) software, respectively. All desired heatmaps were prepared with TBtools1.055 (Chen et al., 2020). The integrated metabolomics and transcriptomic analysis was performed with two-way orthogonal PLS analysis by using the OmicsPLS package of R (Bouhaddani et al., 2016). Additionally, Pearson correlation coefficients were analyzed and only the top 250 pairs of genes and metabolites with correlation >0.50 were selected to construct transcripts to metabolite final network with the igraph packages in R (Csardi and Nepusz, 2006).

## Data Availability

The raw RNA-seq data have been deposited at National Genomic Data Center under the project number PRJCA012977 (https://ngdc.cncb.ac.cn/search/?dbId=andq=PRJCA012977).
