# Supplementary material for: Metabolite profiling and transcriptome analyses provide insight into the regulatory network of graft incompatibility in litchi
Source: Front Genet. 2023 Jan 5;13:1059333. doi: 10.3389/fgene.2022.1059333 (PMC9849251; doi:10.3389/fgene.2022.1059333)
Supplement: Supplementary file 1 [file Table2.DOCX]

Table S2: Mean statistics of sequenced data for graft compatible LY and incompatible YL combinations of litchi

| Sample | Raw reads | Clean reads | Q30 (%) | GC (%) | Total Mapped (%) | Unique Mapped (%) | Multiple Mapped (%) |
| --- | --- | --- | --- | --- | --- | --- | --- |
| LY | 7038200400 | 6973474423 | 92.275 | 43.79 | 82.905 | 78.47 | 4.435 |
| YL | 7182378150 | 7116452465 | 92.145 | 43.825 | 83.68 | 79.165 | 4.515 |
